# Mobile surgical services in primary care in a rural and remote setting: Experience and evidence from Yala, Cross River State, Nigeria

**DOI:** 10.4102/phcfm.v1i1.31

**Published:** 2009-07-28

**Authors:** Emmanuel Monjok, Ekere J. Essien

**Affiliations:** 1 Institute of Community Health, University of Houston, USA

**Keywords:** mobile surgery, sub- Saharan Africa, primary health care, rural health, Nigeria

## Abstract

Surgical conditions account for 11 to 15% of the global burden of disease. Yet, surgical services are very scarce in the rural areas of Nigeria where approximately 60 to 80% of the population resides. Among other basic contributing factors is the shortage of surgical workforce, since Nigeria’s few surgeons practise in the urban centre of the major cities. One way to respond to this acute shortage of surgeons is the training of generalist medical doctors to undertake surgery in rural areas. The introduction of mobile surgical services in rural populations as part of the existing primary health care activities in the Local Government Areas (districts) can reduce surgical morbidity and mortality in Nigeria. This can be done by the generalist physician with training and experience in surgery using local health staff and simple surgical equipment. A number of recommendations are made.

## INTRODUCTION

There is an increasing awareness of the need to increase surgical services at district hospitals and health facilities in the rural populations of sub-Saharan Africa.^1-4^ A conference on increasing access to surgical services in resource-constrained settings in sub-Saharan Africa, organised by Global Health Sciences, University of California at San Francisco and others, has further highlighted this awareness^5^. The World Health Organization (WHO) Global Initiative for Emergency and Essential Surgical Care has also brought to the fore the fact that surgical services have an important role to play as preventive and life saving strategies in public health^6^. Although surgical conditions account for an estimated 11 to 15% of the global burden of disease,^7^ they are not accorded the same priority as other preventive procedures in primary heath care PHC), such as immunisation and prevention of mother to child transmission of HIV.

Surgeons are in extremely short supply in most hospital districts in sub-Saharan Africa and the existing few are mostly stationed at the national or teaching hospitals in major cities. However, most of the surgical cases are to be found in the rural areas where at least 80% of the population resides.^1-7^ To meet this volume of surgical services many African countries rely on non-physicians who are trained to provide surgical services in rural hospitals with good clinical outcome and economic benefits in terms of training cost to the government.^8,9^

Generalist physicians (general practitioners and family physicians) with surgical and obstetric skills are the mainstay of surgical procedures in many sub-Saharan countries, including Nigeria.^1-7^ Most of the medical officers practising in remote centres do not have adequate surgical training to meet the challenges of the volume of surgical cases and procedures at their location. The Nigerian postgraduate medical training programme in general practice/family medicine was designed to bridge this gap and give adequate surgical exposure to residents who will eventually function as gatekeepers in rural and remote communities.

One of the components of PHC is the treatment and care of common health conditions in the community. However, as stated, in Nigeria treatment of common surgical conditions is not usually provided in rural and remote communities by PHC teams. One of the reasons why this does not occur is the severe shortage of surgical skills in the health manpower of district or local government PHC teams in Nigeria. Nigeria is one of the countries in sub-Saharan Africa that does not train non-physicians for surgical services.^8^

One way of increasing access to surgical services in rural and resource-constrained populations is to introduce mobile surgical services (MSS). In this article, the researchers present their experience from Yala Local Government Area (district) in the Cross River State of Nigeria regarding the use of MSS to increase access to surgical services that had not existed previously in the rural and remote communities. This article will attempt to provide some evidence that surgery as it occurs in a large urban and complex traditional system can be comfortably extended to rural and remote communities with the available resources and manpower at the local sites and with little cost to the area’s inhabitants.

### Yala LGA

Yala Local Government Area is one of the 18 LGAs that make up the Cross River State of Nigeria. Nigeria, located in the West African sub-region, is Africa’s most populous nation, with 36 states and a federal capital territory in Abuja. Yala LGA, created in 1991, is one of the 774 LGAs that constitute the Nigerian federation. It has an estimated population of about 600 000 people, of which 90% are living in rural villages. These villages are situated far from each other, with very difficult terrain and a poor network of roads. Since there was no government district hospital in Yala LGA in 1991, the Lutheran Hospital, a non-governmental organisation, has been the provider of hospital services in this LGA. The LGA has 17 health centres (HCs), three of which are comprehensive health centres (CHC). One CHC located at LGA headquarters has 15 beds and a newly built and equipped surgical centre. The other two CHCs, with about 10 hospital beds each, are located in remote communities.

## PROBLEM STATEMENT

One of the researchers (Emmanuel Monjok) was seconded from the Ministry of Health as Community Health Physician/ Primary Health Care Coordinator (Medical Officer of Health) for 12 months to assist the newly created LGA, to set up the PHC department and to manage and deliver the PHC components. Medical officers are not normally employed by the local government service in Cross River State. The categories of health staff in the local government service consist of community health officers, registered nurses, midwives, community health aids, theatre assistants/aids and pharmacy assistants/aids.

In Nigeria, a mobile surgical service (MSS) is not normally a component of PHC. It was incorporated as part of the services to be rendered due to the following reasons:

1. There was a surgical centre with adequate surgical instruments and sterilisation at the LGA headquarters.
2. The rural villages are far from the LGA headquarters where this surgical centre is located. The terrain by road is particularly rough and difficult especially during the rainy season.
3. The surgical centre was newly built and commissioned and was not being utilised.
4. The researcher had previously conducted a situational analysis and report (unpublished) of the 17 HCs and found out that 16 health facilities, in particular the two remote CHCs, were all well equipped for some surgical services. All had a small functional generator to provide electricity at night for a limited period of time.
5. The same researcher had general surgical/obstetric/ anaesthetic skills having been trained for 14 months in a general practice/family medicine residency programme of the National Postgraduate Medical College of Nigeria at the Roman Catholic Medical Missionary of Mary hospital, a rural/peripheral, 250 bed hospital.
6. Said researcher had previous experience with mobile eye services and had coordinated and managed the Roman Catholic Medical Missionary of Mary hospital mobile eye programme with a visiting ophthalmologist from the Netherlands in January/February 1987, January/Febuary 1988 and January/February 1989. The researcher had obtained ophthalmology training as well, as part of the general practice/family medicine residency training.

### The mobile surgical service

The WHO/UNICEF funded immunisation programme which has a mobile component provided the movement logistics for the MSS. The UNICEF van and the local government PHC van were used to convey the MSS team from one HC to another. The movement schedules were distributed to all health facilities on a monthly basis (i.e. a month ahead of the scheduled visit), to allow the chief Health Officers and Registered Nurses to screen the patients and engage in community mobilisation activities. The clan village chiefs also participated in mobilising their communities for PHC and MSS activities. The screened surgical cases were evaluated (by Emmanuel Monjok) before being accepted for the surgery list.

The MSS program was heavily subsidised by the local government council with fees slashed as much as 50%. Major surgeries were not performed during MSS; any major case was referred to the central surgical centre at the local government headquarters. The supplies for the MSS consisted of medical and non-medical items (see Tables 1 and 2 respectively). The surgical conditions, procedures and anaesthetic techniques used are outlined in Table 3.

**TABLE 1:**
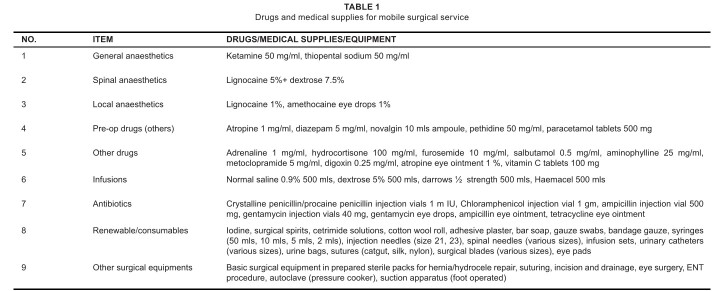
Drugs and medical supplies for mobile surgical service

**TABLE 2:**
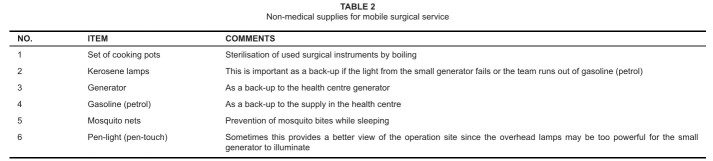
Non-medical supplies for mobile surgical service

**TABLE 3:**
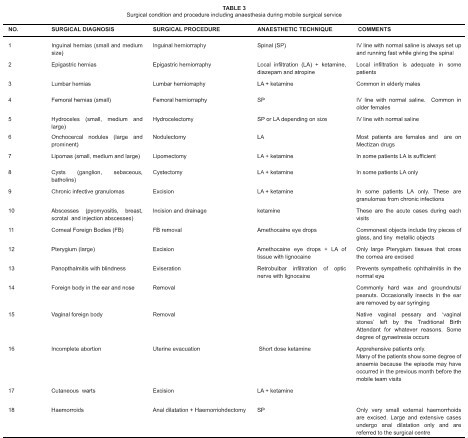
Surgical condition and procedure including anaesthesia during mobile surgical service

## LESSONS LEARNED

1. The health centres were equipped with basic surgical equipments without having the corresponding health manpower.
2. There are many surgical cases in the remote populations but only when services are brought close to patients’ door steps is utilisation enhanced. It is possible to minimise surgical complications with well selected patients (i.e. patients who are unlikely to have complications), good surgical techniques and dedicated nursing care.
3. Rural people should not be under-rated. They are willing to pay for surgical services if within their reach, especially if the fees are subsidised, as was the case in Yala LGA.
4. Community mobilisation is an important tool in increasing surgical access in rural populations, irrespective of the fact that these services are not provided free of charge.
5. The importance of mobile health was appreciated in one community completely cut off from the rest of the LGA by a huge river. Our MSS team (conveyed by canoe) was the first health team to visit this remote community. No surgical services occurred in this community as the HC is a makeshift temporary post in the local primary school (a community programme). An appeal was made to the chairman of the LGA for this isolated community to be provided with a government built HC and that immunisation teams specifically should be specially conveyed to this community in all future health programmes.

### Recommendations

1. The local government service commission in Cross River State should be given the mandate to employ medical officers in their establishment. This ensures continuity of primary medical and health care to the communities in the LGA.
2. The development of a career structure for medical officers’ careers should be entrenched constitutionally by the local government service commission.
3. Medical officers in the employ of the local government service commission should be trained in general practice/family medicine as well as in general preventive medicine and public health.
4. As a long term goal, the National Postgraduate Medical College of Nigeria should introduce a rural and remote medical certification as an added qualification in the speciality of general practice/family medicine for medical doctors interested in rural medicine, as is being championed in Australia.
5. MSS should undergo evaluation and testing in other parts of Nigeria and a national policy for MSS should be formed to ensure continuity and acceptance.
6. MSS teams could also function in health education programmes and health communication programmes as well as increase awareness for HIV testing, counselling and ART therapy in these remote communities.
7. Operational research on MSS is essential for more input and outcome measures.

### Conclusion

Surgical services can be offered to rural populations of remote areas in Nigeria and all other sub-Saharan countries where the surgical workforce is limited. This is feasible through a mobile unit, utilising the static health centres in these communities and the basic surgical instruments and local health staff.

Since PHC and immunisation activities are already well entrenched in the local government health systems, MSS can become an important service in resource-constrained settings. The generalist physician with training in surgery and obstetrics and some basic training in public health can effectively increase the volume of surgical services and reduce surgical and maternal mortality and morbidity.
